# p21WAF1/CIP1 gene transcriptional activation exerts cell growth inhibition and enhances chemosensitivity to cisplatin in lung carcinoma cell

**DOI:** 10.1186/1471-2407-10-632

**Published:** 2010-11-19

**Authors:** Junxia Wei, Jiang Zhao, Min Long, Yuan Han, Xi Wang, Fang Lin, Jihong Ren, Ting He, Huizhong Zhang

**Affiliations:** 1 Department of Clinical Laboratory and Research Center, Tangdu Hospital; Fourth Military Medical University; Xi'an, China; 2 Department of Orthopaedics, Xi'an Railway Central Hospital; Xi'an, China

## Abstract

**Background:**

Non-small-cell lung carcinomas (NSCLCs) exhibit poor prognosis and are usually resistant to conventional chemotherapy. Absence of p21WAF1/CIP1, a cyclin-dependent kinase (cdk) inhibitor, has been linked to drug resistance in many in vitro cellular models. RNA activation (RNAa) is a transcriptional activation phenomena guided by double-strand RNA (dsRNA) targeting promoter region of target gene.

**Methods:**

In this study, we explored the effect of up-regulation of p21 gene expression on drug-resistance in A549 non-small-cell lung carcinoma cells by transfecting the dsRNA targeting the promoter region of p21 into A549 cells.

**Results:**

Enhanced p21 expression was observed in A549 cells after transfection of dsRNA, which was correlated with a significant growth inhibition and enhancement of chemosensitivity to cisplatin in A549 cells in vitro. Moreover, in vivo experiment showed that saRNA targeting the promoter region of p21 could significantly inhibit A549 xenograft tumor growth.

**Conclusions:**

These results indicate that p21 plays a role in lung cancer drug-resistance process. In addition, this study also provides evidence for the usage of saRNA as a therapeutic option for up-regulating lower-expression genes in lung cancer.

## Background

Lung cancer is the most common cause of cancer mortality worldwide. Non-small-cell lung carcinomas (NSCLCs), which represent around 80% of lung tumors, exhibit poor prognosis and are usually resistant to conventional chemotherapy. Cisplatin is one of the most potent anticancer agents, displaying significant clinical activity against a variety of solid tumors. The most effective systemic chemotherapy for non-small cell lung cancer (NSCLC) was cisplatin-based combination treatment. Unfortunately, the outcome of cisplatin therapy on NSCLC seems to be unsatisfactory. The use of cisplatin in cancer chemotherapy is limited by acquired or intrinsic resistance of cells to the drug. The cytotoxicity of cisplatin is believed mainly due to interaction with DNA, forming inter-and intra-strand adducts, hindering both RNA transcription and DNA replication, leading to cell cycle arrest and apoptosis. Numerous cellular mechanisms potentially contributing to clinical cisplatin resistance have been proposed, including changes in cellular drug accumulation, detoxification of the drug, inhibition of apoptosis and repair of the DNA adducts but the precise mechanisms are still need to be validated. It has been reported that P21 expression level is involved in the resistant phenotype of this drug [1-4].

p21WAF1/CIP1 (p21) is a well-characterized cyclin-dependent kinase (cdk) inhibitor that belongs to the Cip/Kip family of cdk inhibitors. It mainly inhibits the activity of cyclin/cdk2 complexes and negatively modulates cell cycle progression [3-6]. Loss or inactivation of p21 is seen clinically in primary solid tumors and related with poor prognosis of these tumors [7,8]. Additionally, there is a growing body of evidence suggesting that functional loss of p21 can mediate a drug-resistance phenotype in tumor therapy [9,10].

RNA-induced gene activation is a transcriptional gene activation phenomenon specifically induced by double small RNA (dsRNA) molecule targeting gene promoter regions. This phenomenon was termed RNAa and the dsRNA molecules were designated small activating RNAs (saRNAs). By targeting gene promoter regions, saRNAs induce the demethylation of histone, leading to transcriptional gene activation. It has been demonstrated that saRNA could inhibit cell proliferation and viability via up-regulation of p21 and E-cadherin in human bladder cancer cells [11-13]. Since saRNAs offer a practical and cost-effective approach to activate gene expression, it may be additional method except for ectopic expression in enhancing expression of targeted genes.

In this study, we explored the effect of up-regulation of p21 gene expression on drug-resistance in A549 non-small-cell lung carcinoma cells by transfecting the saRNA targeting the promoter region of p21 into A549 cells. We observed activation of p21 expression in A549 lung carcinoma cells after transfection of saRNA. The enhanced p21 expression was correlated with a significant growth inhibition and enhancement of chemosensitivity to cisplatin in A549 cells in vitro and vivo. These results provide evidence of an additional therapeutic strategy for lung cancer therapy especially for chemoresisitance lung carcinomas.

## Methods

### Design and preparation of dsRNA

saRNA targeting the promoter of p21 at position-322 relative to the transcription start site was termed as dsP21-322 and designed as previously described [9]. Scramble dsRNA with the following sequence: S, 5'-UUCUCCGAACGUGUCACGU [dT][dT]-3'; AS, 5'-ACGUGACACGUUCGGAGAA[dT][dT]-3' was also synthesized and used as control. Synthetic dsRNAs were manufactured by Genepharma Inc (Shanghai, China).

### Cell culture and transfection

Human lung carcinoma cells (A549) were cultured in Dulbecco's modified Eagle's medium (DMEM, Invitrogen) supplemented with 10% fetal bovine serum and penicillin (100 Units/ml)/streptomycin(0.1 mg/ml) in 5% CO_2 _incubator at 37°C. Cells were seeded into six-well plates with growth medium at a density of 0.8 × 10^5 ^cells/well respectively and cultured overnight to (30-50)% confluence prior to transfection. Cells were then transfected with 100 pmol/well of dsP21-322 or scramble dsRNA, respectively, using the LipofectamineTM2000 reagent (Invitrogen, USA) according to the manufacturer's protocols.

### RNA isolation and semi-quantitative RT-PCR

Total RNAs were extracted from dsP21-322, scramble dsRNA and mock transfected A549 cells by using TRIzol reagent according to the manufacturer's instructions. Complementary DNA (cDNA) was generated from total RNA by reverse transcription using moloney murine leukemia virus (M-MLV). PCR amplification of the cDNA was performed in a reaction mixture with a final volume of 30 μL containing 2 μL of 4 × dNTPs, one unit of Taq DNA polymerase, and 10 mmol/L of each paired primer specific to p21 gene. The primers used for RT-PCR of p21 were forward primer, 5'-TTGATTAGCAGCGGAACA-3' and reverse primer, 5'-TACAGTCTAGGTGGAGAAACG-3'.

### Western blotting

The cells from experiment group and control groups were harvested and washed with PBS (pH 7.4) twice and resuspended in lysis buffer (1 mM dithiothreitol, 0.125 mM EDTA, 5% glycerol, 1 mM phenylmethylsulfonylfluoride, 1 μg/mL leupeptin, 1 μg/mL pepstatin, 1 μg/mL aprotinin, 1% Triton X-100 in 12.5 mM Tris-HCl buffer, pH 7.0) on ice. The cell extracts were clarified by centrifugation and the protein concentrations were determined by using the Bio-Rad protein assay kit (Bio-Rad, Hercules, CA). Each protein extract (25 μg) was electrophoresed on a 12% SDS-polyacrylamide gel, transferred to PVDF membrane in a buffer containing 25 mM Tris-HCl (pH 8.3), 192 mM glycine, 20% (v/v) methanol, and blocked in 5% (w/v) skimmed milk in Tris buffered saline-Tween 20 (0.1% by volume, TBST) for 1 hour at room temperature, and probed with specific primary antibodies overnight at 4°C. Then primary antibodies were removed and the blots were extensively washed with TBST for three times. Blots were then incubated for an hour at room temperature with the secondary antibodies (goat anti-rabbit/mouse antibody coupled to horseradish peroxidase, 1:3000 dilution) in 1% (w/v) skimmed milk dissolved in TBST. Following removal of the secondary antibody, blots were extensively washed as above for an hour and developed using the Enhanced Chemiluminescence Kit (NENTM Life Science Products Inc, Boston, MA). The primary antibodies used in this experiment for western blotting analysis were anti-p21 (1:100, Santa Cruz) and anti-β-actin (1:500, Sigma) antibody.

### 2.5 3-(4, 5-Dimethyl-2-thiazolyl)-2, 5-diphenyl-2Htetrazolium bromide (MTT) assay

MTT assay was performed to assess the effect of p21 expression on cell proliferation. Transiently transfected lung carcinoma cells were plated in 96-well plate at a density of 3.0 × 10^3 ^cells/well for proliferation assay. Then for 5 days, every 24 h a batch of cells were stained with 20 μl sterile MTT dye (5 mg/ml; Sigma, USA) at 37°C for 4 h, then culture medium was removed and 150 μl of DMSO was added and thoroughly mixed in for 10 min. Spectrometric absorbance at 490 nm was measured by using a microplate reader. All experiments were performed in triplicate.

### Colony formation assay

Approximately 0.5 × 10^3 ^A549 cells transiently transfected with dsP21-322, scramble dsRNA and mock were plated in 100-mm culture dishes, respectively. After 18 days, cells were fixed with methanol and stained with 0.1% crystal violet. Visible colonies were manually counted.

### Flow cytometric analysis of apoptosis

An annexin V-fluorescein isothiocyanate apoptosis detection kit (Zymed, USA) was used to detect cell apoptosis. Approximately 1 × 10^6 ^A549 cells transiently transfected with dsP21-322, scramble dsRNA and mock, respectively, were harvested and analyzed by Flow Cytometry (BD, USA).

### In vitro chemosensitivity assay

The dsP21-322, scramble dsRNA and mock transfected A549 cells were seeded in 96-well plate at a density of 5 × 10^4 ^cells/well. The cells were then treated with 5 μg/ml cisplatin for 48 h.Then, 20 μl of MTT stock solution (5 mg/ml) was added to each well, and the cells were incubated at 37°C for 4 h. The supernatant was replaced with DMSO to dissolve formazan production. The A490 nm values were assayed in a microplate reader. The ratio of the absorbance of treated cells relative to that of the control cells was calculated and expressed as a percentage of cell viability. The mean of three parallel samples was calculated. Experiments were performed in triplicate and standard deviations were calculated based on the average of three experiments.

### In vivo chemosensitivity assay

A549 cells (1 × 10^6^) were injected subcutaneously into the right posterior limb of BALB/c nude mice (4-6 weeks old). When palpable tumors (about 100-130 mm^3^) arose within 16-21 days, mice were randomized to treatment and control groups. Three groups (five mice each) received intratumoral injections of mixture of 30 μg of LipofectamineTM2000-encapsulated dsP21-322, scramble dsRNA and PBS respectively, every 3 days for 3 weeks. The other two groups received intratumoral injection of PBS combined with cisplatin or dsP21-322 combined with 5 mg/kg cisplatin, individually, every 3 days for 3 weeks. Tumor growth was monitored by caliper-measuring two perpendicular tumor diameters every 3 days, and the volume of the tumor was calculated from the formula: V = (width^2 ^× length × 0.5). At the end of the experiment, tumor weight was assessed by sacrificing the mice, and by removing and weighing the tumor. Animal experiments in this study were carried out in accordance with the medicine institutional guidelines of Fourth Military Medical University.

### Immunohistochemistry of tumors

The sections of the tumor tissues embedded in paraffin were stained using mouse anti-p21 antibody (Santa Cruz) at 1:50 dilution overnight at 4°C. After brief washing, all slides were stained and visualized with a Histofine SAB-PO(M) kit (Nichirei, Tokyo, Japan) according to the manufacturer's instructions.

### Statistical analysis

Results were expressed as Means ± standard deviation (SD). Statistical analyses were performed using SPSS statistical software. Student's t-test and one-way analysis of variance (ANOVA) followed by Dunnett's multiple comparison tests were adopted. Values of p < 0.05 were considered as significant and indicated by asterisks in the figures.

## Results

### P21 was up-regulated by saRNA in A549 cell line

**Figure 1:**
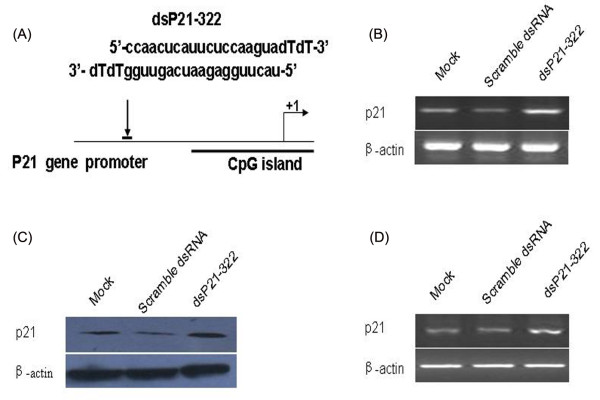
**dsP21-322 induces p21 gene expression in cell lines**. (A) A schematic representation dsP21-322 position and sequence on the promoter region of p21 gene. (B) Semiquantitative RT-PCR result showing elevated p21 mRNA expression in dsP21-322 transfected A549 cells. (C) Western blot analysis showing increased p21 protein expression in dsP21-322 transfected A549 cells, and the results were normalized to β-actin. (D) Semiquantitative RT-PCR result showing elevated p21 mRNA expression in dsP21-322 transfected Saos2 cells.

As an initial test of our study, we transfected saRNA targeting the p21 gene promoter at position-322 relative to the transcription start site (dsP21-322) into A549 cells for 72 h (Figure 1A). Semiquantitative RT-PCR and Western blotting results showed that both mRNA and protein expressions of p21 were elevated in dsP21-322 transfected A549 cells (Figure 1B, C), comparing with mock or scramble dsRNA transfected group. To further explore whether up-regulation of the p21 mRNA is independent of p53 protein expression, we detected the effect of dsP21-322 on p53 null Saos2 cells. As shown in Figure 1D, dsP21-322 was able to elevate the p21 mRNA level in p53 null cells.

### Lung carcinoma cell proliferation and colony formation were inhibited by p21 up-regulation in vitro

**Figure 2:**
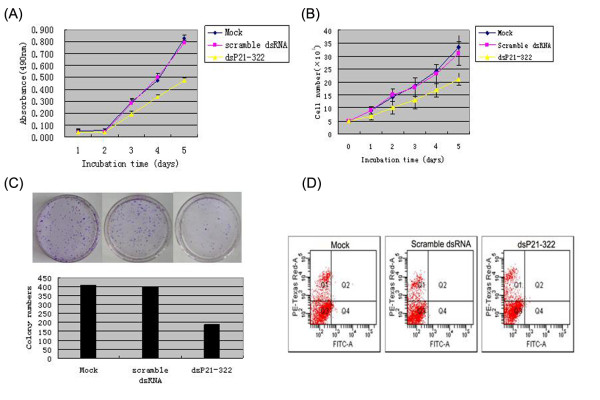
**Effects of dsP21-322 on cell proliferation, colony formation and apoptosis**. (A) Mock, scramble dsRNA and dsP21-322 transiently transfected cells were measured by MTT assay for the cell proliferation. (B) Mock, scramble dsRNA and dsP21-322 transfected A549 cells were treated with cisplatin and numbers of viable cells were counted by Trypan blue exclusion. (C) The mock, scramble dsRNA and dsP21-322 transfected A549 cells were seeded onto 100-mm culture dishes at a concentration of 0.5 × 10^3 ^cells and cultured for 17 days. Colony formation of dsP21-322 transfected A549 cells was inhibited obviously. (D) Apoptotic analysis by Flow Cytometry showing negative results in all of mock, scramble dsRNA and dsP21-322 transfected A549 cells.

An important characteristic of tumor cells is their increased proliferative capability, which is often caused by impaired regulation of the cell cycle. It has been reported that p21 can regulate the cell cycle process by binding and inhibiting cyclin-dependent kinases, so we examined the effect of p21 transcriptional activation on the proliferation of A549 cells in vitro. In this experiment, cell proliferation was monitored by MTT assay daily for 5 days. The cell growth curve showed that proliferation of dsP21-322 transfected A549 cells was significantly inhibited in a time-dependent manner, while scramble dsRNA transfected A549 cells showed no significant inhibition of the proliferation (Figure 2A). Trypan blue exclusion results also showed that the cell proliferation of dsP21-322 transfected A549 cells was inhibited (Figure 2B). In colony formation assay, as expected from the results of MTT assay, the numbers of colonies were obviously decreased in dsP21-322 transfected A549 cells compared with mock and scramble dsRNA transfected A549 cells (Figure 2C). Although up-regulation of p21 could inhibit proliferation and colony formation in A549 cells, there was no obviously difference in apoptotic rates between dsP21-322 transfected cells and scramble dsRNA transfected cells detected by Flow cytometric analysis (Figure 2D). All these results suggested that up-regulation of p21 gene expression by saRNA could lead to significant inhibition of lung carcinoma cell proliferation in vitro.

### The effect of up-regulation of p21 gene expression on the cell cycle

**Figure 3:**
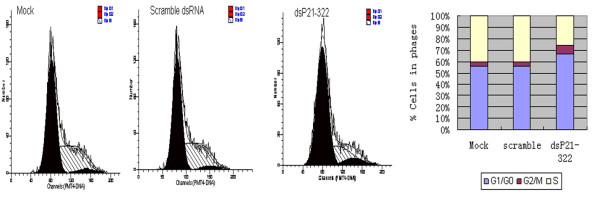
**Cell cycle analysis of dsP21-322 transfected A549 cells by Flow Cytometry**. It was shown that percentage of the cells in G1/G0 phase was increased in dsP21-322 transfected cell (66.92%) compared with that in the mock and scramble dsRNA transfected cells (55.87% and 56.23% respectively). Percentage of cells in S phase was decreased to 25.67% compared with that in the mock and scramble dsRNA transfected cells (40.28% and 40.15%, respectively)

It has been shown that overexpression of p21 results in G1-, G2-, or S-phase arrest [14-16], we explored the cell cycle changes in p21 up-regulated A549 cells. Cell cycle analysis by flow cytometry method showed that the percentage of the cells in G1/G0 phase was increased in dsP21-322 transfected cells (66.92%) compared with that in the mock and scramble dsRNA transfected cells (55.87% and 56.23% respectively) (Figure 3). Nevertheless, percentage of cells in S phase in dsP21-322 transfected cells decreased to 25.67% compared with that in mock and scramble dsRNA transfected cells (40.28% and 40.15%, respectively). These results indicated that up-regulation of p21 gene expression mainly blocked A549 cells in G1/G0, which might lead to proliferation inhibition of the cells.

### The specific up-regulation of p21 gene expression enhances cisplatin cytotoxicity in vitro

**Figure 4:**
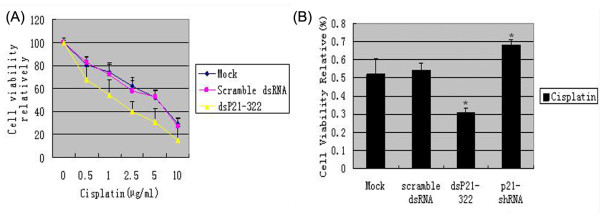
**Toxicity assay of cisplatin on dsP21-322 transfected A549 cells**. (A) Human lung cancer A549 cells transfected with mock, scramble dsRNA or dsP21-322 was treated by cisplatin with concentrations ranging from 0.5 μg/ml to 10 μg/ml for 48 h. The IC_50 _of dsP21-322 treated cells was decreased to 1.23 μg/ml compared with those of mock and scramble dsRNA transfected groups (4.15 μg/ml and 3.84 μg/ml respectively). (B) Transiently transfected A549 cells were treated with 5 μg/ml cisplatin, drug sensitivity analysis was performed with MTT assays. Data represent the mean + SE of three independent experiments. * p < 0.05.

Previous study showed that p21-negative cells were defective in nucleotide excision repair, which has been suggested to increase sensitivity to certain chemotherapeutic drugs [17]. So we next explored whether the saRNA-mediated up-regulation of p21 gene expression could affect the sensitivity of A549 cells to the antitumor agent cisplatin. We treated the scramble dsRNA or dsP21-322 transfected A549 cells by cisplatin with concentrations ranging from 0.5 μg/ml to 10 μg/ml for 48 h. The relative cell viability at different cisplatin concentrations were made to calculate the IC_50_. The results showed that the IC_50 _of dsP21-322 transfected cells was decreased to 1.23 μg/ml compared with those of mock and scramble dsRNA transfected groups (4.15 μg/ml and 3.84 μg/ml respectively) (Figure 4A). To further confirm the role of p21 in sensitivity of A549 cells to cisplatin, the endogeneous p21 expression was knocked down by p21-shRNA, the cytotoxicity of the cisplatin at the concentration of 5 μg/ml was measured with MTT assays. As shown in Figure 4B, the inhibition rates of dsP21-322 treated cells increased statistically. While in cells with p21 expression silenced by shRNA, the chemosensitivity of A549 cells to cisplatin was decreased (p < 0.05).

### The effect of P21 up-regulation on chemosensitivity to cisplatin in vivo

**Figure 5:**
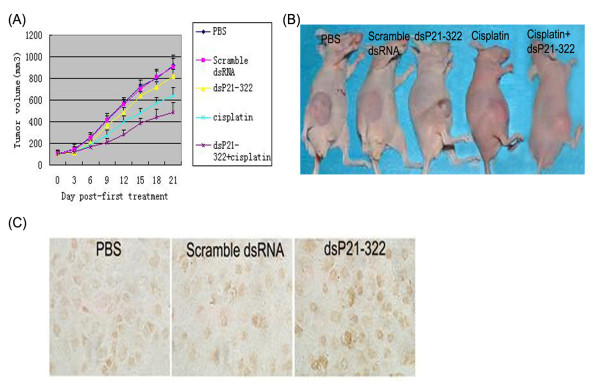
**Up-regulation of p21 gene expression enhanced cisplatin cytotoxicity in vivo**. (A and B) Aliquots of 1.0 × 10^6 ^A549 cells were suspended in 1:1 PBS subcutaneously inoculated into the right flank of each mouse. When palpable tumors were observed, mice were stochastically assigned to 5 groups (n = 5). Mice were received PBS, scramble dsRNA, dsP21-322, cisplatin, or mixture of dsP21-322 and cisplatin intratumorally every 3 days for three weeks. During three weeks treatment, tumor volume was monitored and tumor growth was curved. (C) Expressions of p21 in tumor tissues treated with dsP21-322 were assessed by immunohistochemical staining.

In view of these findings in vitro, we further tested the efficacy of dsP21-322 as an in vivo chemosensitivity strategy in nude mouse xenograft model. When palpable tumors arose in the right flank of mice, the mice received PBS, scramble dsRNA, dsP21-322, PBS combined with cisplatin or dsP21-322 combined with cisplatin intratumorally every 3 days until the end of the experiment. The tumor size was monitored every 3 days for three weeks. As shown in Figure 5A,B, cisplatin treatment combining with dsP21-322 inhibited the xenograft tumor growth significantly compared with the group treated with cisplatin combining with PBS (p < 0.05). In addition, we detected the expression of p21 in the formalin embedded tumor tissue using anti-p21 antibody. As shown in Figure 5C, the expression of p21 proteins were increased in dsP21-322 treated tumor tissues. Hence, p21 saRNA along with chemotherapeutic cisplatin could produce a synergistic cytotoxicity effect on the cell proliferation of lung carcinoma in vivo.

## Discussion

Lung cancer is considered usually to acquire resistance to chemotherapy during multiple courses of therapy, which leads to poor prognosis, compared with other types of human malignancies. Thus, attempts at improving the survival of patients affected by this disease depend largely on strategies targeting development of tumor cell resistance to chemotherapy drugs, which cannot be rationally planned without a detailed knowledge of the mechanisms underlying this phenomenon. Searching for molecular targets participating in the process of drug-resistance and utilizing these targets to oppose drug-resistance in chemotherapy will be beneficial to the clinical therapy. There is evidence that alteration of CDK inhibitors in cancer may affect the response to chemotherapeutic treatment. Loss expression of p21 has been linked to drug-resistance in many in vitro cellular models. However, to date, evidence about the relationship between this CDK inhibitor and lung carcinoma drug-resistance has been lacking.

It has been reported that genetic and epigenetic abnormalities can induce lower expression of p21, which is linked to chemoresistance in many in vitro cellular models [18]. Colon cancer cells with deletions of p21WAF1/CIP1 showed abnormal response to treatment with doxorubicin, which is due to abnormal block to G2 decreases undergoing mitosis of cell [19]. It was also demonstrated that forced overexpression of p21WAF1/CIP1 in osteosarcoma cells increased sensitivity to chemotherapeutic agents and leaded to G1 and G2/M arrest [20,21]. These results indicate that deletion or lower expression of p21 is involved in drug-resistance. Various mechanisms exist to regulate the levels of p21 in a cell including transcriptional regulation, epigenetic silencing, mRNA stability, and ubiquitin-dependent and-independent degradation of the protein [22]. The dsRNA used in RNAa study was designed by closely following rational siRNA design rules and avoided CpG-rich islands. These characteristics may direct the modification of histone and further the activation or silencing of target gene [11]. Cisplatin have been used as first-line therapy to treat lung carcinoma, but its curative effect is far from satisfactory. Thus, in order to improve the prognosis of patients with type of refractory cancer, it is necessary to identify and target genes which conduce to the treatment of lung carcinoma, such as enhancement of conventional chemotherapy.

In this study, we elevated the expression of p21 in lung carcinoma A549 cells by using saRNA targeting the promoter region of p21, which has been demonstrated to transcriptionally activate the expression of p21 gene. We detected up-regulation of p21 after tranfection of saRNA compared with scrambled dsRNA in A549 cells, the results showed that the expression of p21 could be increased in lung cancer cells by saRNA transfection.

To explore the phenotype changes induced by p21 up-regulation in A549 cells, we detected the proliferation, colony formation, apoptosis and cell cycle change of saRNA transfected cells. The results showed that up-regulation of p21 by transcriptional activation inhibited the proliferation and colony formation of lung cancer cells. Cell cycle analysis showed that endogenous p21 up-regulation induced cell accumulation both in the G1/G0 phase in lung cancer cells, which leads to proliferation inhibition of lung cancer cells, but there was no apoptosis cells detected after dsP21-322 transfection. It was reported that p21 plays dual roles as both as pro and anti-apoptotic gene. Whether p21 exhibits pro or anti-apoptotic effects is likely to depend on the specific cellular context [23]. In our study, we did not detect obviously difference in apoptotic rates between dsP21-322 transfected cells and scramble dsRNA transfected cells. These data indicated that activation of p21 expression inhibited the proliferation and enhanced chemosensitivity to cisplatin by process unrelated with apoptotic pathway. Then, we detected the chemosensitivity of saRNA transfected A549 cells to cisplatin in vitro, the results showed that up-regulation of p21 obviously enhanced the sensitivity of A549 cells to cisplatin in vitro. Although we did not find the precise mechanism of this phenomenon, further studies are needed to clarify the accrual role of p21 up-regulation to chemosensitivity of cisplatin. We observed that chemosensitivity of dsP21-322 transfected A549 cells to paclitaxel also increased comparing with control group. Chemotherapy with combination of platinum drug and paclitaxel is a relative effective strategy in non-small-cell lung carcinomas therapy. Enhancement of chemosensitivity to both cisplatin and paclitaxel (Additional file 1, Figure S1) indicates that it is due to their crossing in the signal pathways for the chemotherapeutic effect. Since the up-regulation of p21 gene expression exerts profound effects on cell growth and enhances chemosensitivity to cisplatin, we explored the therapeutic role of p21 in combination with cisplatin in animal models. We observed that tumor growth was inhibited more obviously in the group treated with dsP21-322 combining with cisplatin than those treated with PBS or scramble dsRNA with cisplatin. In this study, as reported on other human malignancies, results from chemosensitivity tests showed that the RNAa-mediated up-regulation of p21 gene expression synergistically enhanced the cytotoxicity of cisplatin both in vitro and in vivo, which made us believe that cisplatin chemotherapy could be more effective in combination with RNAa-mediated up-regulation of p21 gene expression.

## Conclusion

In summary, this study demonstrates that up-regulating expression of p21 in lung cancer by RNAa technique can inhibit proliferation, enhance chemotherapeutic sensitivity to cisplatin in vitro and vivo, which may significantly contribute to therapy of lung cancer, especially for drug-resistance tumor therapy.

## Competing interests

All authors declare that they have no financial or personal relationships with other people or organizations that could inappropriately influence (bias) their work.

## Authors' contributions

YH and TH carried out the cellular studies, XW and JHR carried out the animal model studies, FL carried out the immunoassays. JXW, JZ and ML participated in the design of the study, performed the statistical analysis and drafted the manuscript. HZZ conceived of the study, and participated in its design and coordination. All authors read and approved the final manuscript.

## Pre-publication history

The pre-publication history for this paper can be accessed here:

http://www.biomedcentral.com/1471-2407/10/632/prepub

## Supplementary Material

Additional file 1 **Toxicity assay of paclitaxel on dsP21-322 transfected A549 cells. **The IC_50 _of dsP21-322 treated cells was decreased to 0.17 μg/ml compared with mock or scramble dsRNA transfected group (0.29 μg/ml and 0.30 μg/ml respectively) Click here for file
