# Supplementary figures and images for: p21WAF1/CIP1 gene transcriptional activation exerts cell growth inhibition and enhances chemosensitivity to cisplatin in lung carcinoma cell
Source: BMC Cancer. 2010 Nov 19;10:632. doi: 10.1186/1471-2407-10-632 (PMC2995802; doi:10.1186/1471-2407-10-632)

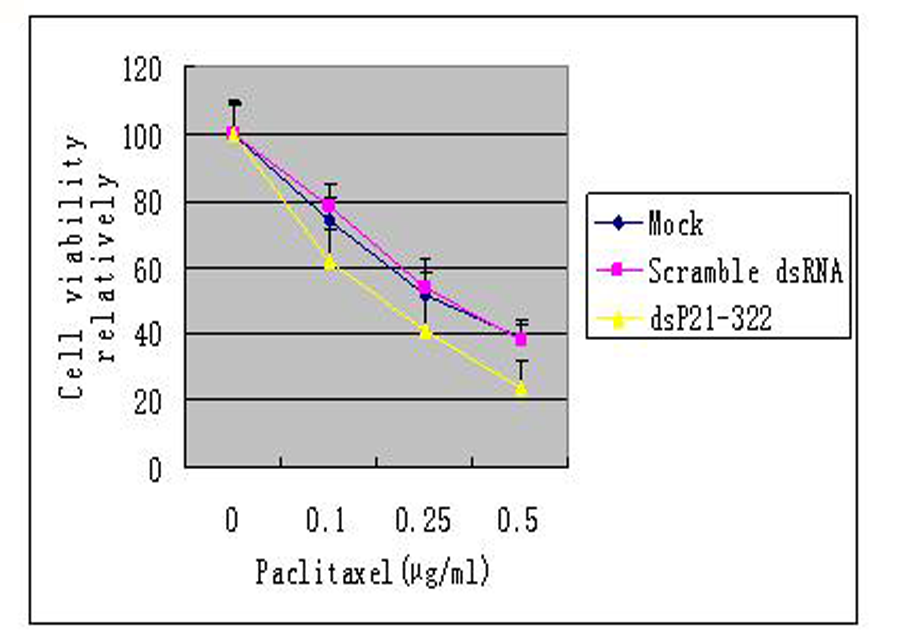

Supplement: Additional file 1 — Toxicity assay of paclitaxel on dsP21-322 transfected A549 cells. The IC50 of dsP21-322 treated cells was decreased to 0.17 μg/ml compared with mock or scramble dsRNA transfected group (0.29 μg/ml and 0.30 μg/ml respectively) [file 1471-2407-10-632-S1.TIFF]
